# Computational Fluid Dynamics as an Engineering Tool for the Reconstruction of Hemodynamics after Carotid Artery Stenosis Operation: A Case Study

**DOI:** 10.3390/medicina54030042

**Published:** 2018-06-01

**Authors:** Andrzej Polanczyk, Michal Podgorski, Tomasz Wozniak, Ludomir Stefanczyk, Michal Strzelecki

**Affiliations:** 1 The Main School of Fire Service, Faculty of Fire Safety Engineering, 01-629 Warsaw, Poland; 2 Department of Angiology, Interfaculty Chair of Anatomy and Histology, Medical University of Lodz, 90-136 Lodz, Poland; michal.podgorski@umed.lodz.pl; 3 Medical Electronics Division, Institute of Electronics, Lodz University of Technology, 90-924 Lodz, Poland; tomasz.wozniak@p.lodz.pl (T.W.); michal.strzelecki@p.lodz.pl (M.S.); 4 Department of Radiology and Diagnostic Imaging, Medical University of Lodz, 90-153 Lodz, Poland; ludomir.stefanczyk@umed.lodz.pl

**Keywords:** brain ischemic stroke, blood hemodynamics reconstruction, artery stenosis, CT-perfusion

## Abstract

*Background and objectives:* Brain ischemic stroke is caused by impaired or absolutely blocked blood flow into the brain regions. Despite the large number of possible origins, there is no general strategy for preventive treatment. In this paper, we aimed to predict the hemodynamics in a patient who experienced a critical stenosis operation in the carotid artery. This is a unique study where we used medical data together with the computational fluid (CFD) technique not to plan the surgery, but to predict its outcome. *Materials and Methods:* AngioCT data and blood perfusion of brain tissue (CT-perfusion) together with CFD technique were applied for stroke formation reconstruction in different clinical conditions. With the use of self-made semiautomatic algorithm for image processing and 3DDoctror software, 3D-vascular geometries before and after surgical intervention were reconstructed. As the paper is focused on the analysis of stroke appearance, apparent stroke was simulated as higher and lower pressure values in the cranial part due to different outcomes of the surgical intervention. This allowed to investigate the influence of spatial configuration and pressure values on blood perfusion in the analyzed circulatory system. *Results:* Application of CFD simulations for blood flow reconstruction for clinical conditions in the circulatory system accomplished on average 98.5% and 98.7% accuracy for CFD results compared to US-Doppler before and after surgical intervention, respectively. Meanwhile, CFD results compared to CT-perfusion indicated an average 89.7% and 92.8% accuracy before and after surgical intervention, respectively. Thus, the CFD is a reliable approach for predicting the patient hemodynamics, as it was confirmed by postoperative data. *Conclusions:* Our study indicated that the application of CFD simulations for blood flow reconstruction for clinical conditions in circulatory system reached 98% and 90% accuracy for US-Doppler and CT-perfusion, respectively. Therefore, the proposed method might be used as a tool for reconstruction of specific patients’ hemodynamics after operation of critical stenosis in the carotid artery. However, further studies are necessary to confirm its usefulness in clinical practice.

## 1. Introduction

Brain ischemic stroke is one of the leading causes of morbidity and mortality in developed countries [1]. In this condition, blood inflow is impaired or absolutely blocked due to an occlusion of the supplying artery [2]. The other type of stroke is hemorrhagic, which develops due to cerebral vessels rupture. Nevertheless, it accounts for only about 10% of all strokes [3,4]. Despite the stroke pathophysiology, it results in irreversible damage to neurons and subsequent major neurological deficits [5].

One of the dominating causes of ischemic stroke are atherosclerosis and its complications [6]. Atherosclerosis is a term referring to a deposition of cholesterol in the arterial wall and associated inflammatory process in the place of atherosclerotic plaque formation [7]. Division of the common carotid artery (CCA) into external and internal carotid artery is especially predisposed to being affected by atherosclerosis due to turbulent blood flow and the associated share stress to the vessel’s wall [8].

Atherosclerosis is a prolonged process that progressively leads to a critical stenosis (greater than 705) and finally to complete occlusion of the artery. On the other hand, some plaques are unstable and may “fracture” leading to rapid thrombus formation and severe ischemia of the upstream region [9]. In these cases, physicians may attempt to restore the patency by surgical or endovascular approach. Nevertheless, the final outcome is not guaranteed and depends on multiple factors e.g., hemodynamics in the operated region [10].

Medical imaging techniques allow to evaluate the morphology of the stenotic artery (ultrasound or CT/MRI angiography) [11] and even flow conditions in the place of the narrowing (Doppler Ultrasound) [12]. However, none of the imaging modalities allow the prediction of what would be the hemodynamic effect of the surgical intervention [13]. The advances in processing of medical data allows to provide realistic in vivo conditions for patient specific analysis (such as reliable anatomical 3D geometries of human cardiac system and initial hemodynamic conditions) [14]. Thus, the stroke management may be improved by a specific therapy and/or an application of advanced prognostic tools, such as computer simulations [15].

Numerical methods require importing imaging data e.g., AngioCT, MRI or Angiography [16]. If the character of the flow has to be evaluated, information from Doppler Ultrasound is mandatory [17]. The Computational Fluid Dynamics (CFD) technique is a useful mathematical tool for hemodynamic investigation [18]. It enables assessment of the diseases severity and improves planning of the reconstructive surgery [19]. Preoperatively created 3D vascular models can be used to predict the hemodynamics and optimize the surgery plans [20].

With the application of the CFD technique, we aim to predict the hemodynamics in a patient who experienced an operation on critical stenosis in the carotid artery. This is a unique study where we use medical data not to plan the surgery but to predict its outcome. The accuracy of the proposed approach will be demonstrated and the reliability of obtained results will be verified with postoperative data.

The paper is organized as follows: in Section 2 medical data, mathematical model and its verification was applied. Section 3 presents the results directed in the mathematical description of blood flow through the defined domains. In Section 4 a discussion was proposed while Section 5 concludes the paper.

## 2. Experimental Section

### 2.1. Medical Data

This is a case study of a 68 year old female who was diagnosed and treated due to a critical stenosis of the left internal carotid artery (stenosis of about 90% of lumen). She presented with a Transient Ischemic Attack (TIA) and due to her symptoms, she underwent AngioCT of the head and neck (GE Light-Speed 64 VCT; GE Healthcare, Fairfield, CT, USA).

**Figure 1:**
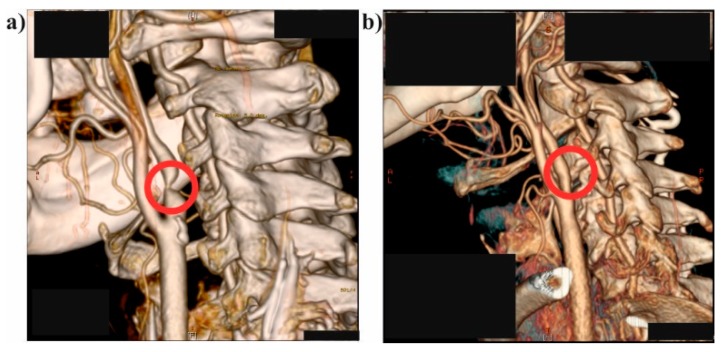
Medical data for the analyzed patient: (**a**) a 3D reconstruction of the left internal carotid artery with critical stenosis (before surgical intervention) (red circle indicates the place of narrowing); (**b**) a 3D reconstruction of the left internal carotid artery without critical stenosis (after surgical intervention) (red circle indicates the place after surgical intervention).

Stenosis of the left internal carotid artery and concomitant disturbances in the left hemisphere perfusion were recognized. Patient was referred to the Doppler Ultrasound (US-Doppler) study (GE Vivid 7, GE Healthcare, Fairfield, CT, USA) to assess hemodynamics in the stenotic region. Critical stenosis (Figure 1a) was diagnosed and due to persisting symptoms, the patient had a balloon angioplasty of the narrowed part of the vessel (Figure 1b).

**Figure 2:**
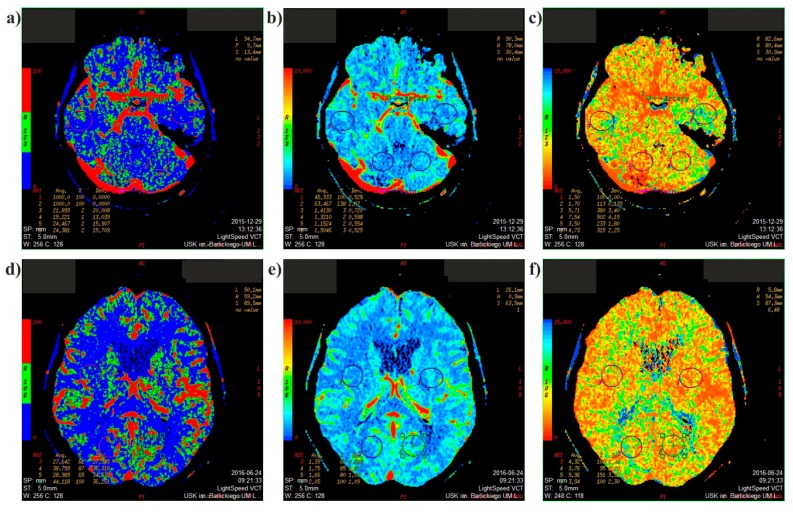
CT-perfusion after surgical intervention, axial scans: (**a**) blood flow—before; (**b**) blood volume—before; (**c**) mean transient time—before; (**d**) blood flow—after; (**e**) blood volume—after; (**f**) mean transient time—after.

For the better evaluation of ischemic area, CT-perfusion study was performed (Figure 2). This is a fine method that evaluates the temporal changes in brain tissue density following contrast administration. The chronological changes in tissue density reflects the nature of tissue vascularity. In ischemic stroke, this technique enables differentiation of irrevocably damaged infarcted brain (the infarct core) from salvageable ischemic brain tissue (the penumbra). This is vital when qualifying for treatment (thrombolysis or clot retrieval). From the CT-perfusion study, the following parameters were analyzed before (Figure 2a–c) and after (Figure 2d–f) surgical intervention: blood flow, blood volume, mean transient time.

To control the surgery effect, another AngioCT with CT-perfusion study and US-Doppler examination were performed after the intervention. US-Doppler records were analyzed to extract velocity profiles as a function of time including one whole cardiac cycle for the purpose of computer simulations. After that, nine velocity profiles were prepared, one as an inlet boundary condition and eight for the verification of outlet conditions, as previously described [21]. The study protocol was approved by the local ethics committee on the Medical University of Lodz (approval no.: RNN/126/07/KE).

### 2.2. Mathematical Model

**Figure 3:**
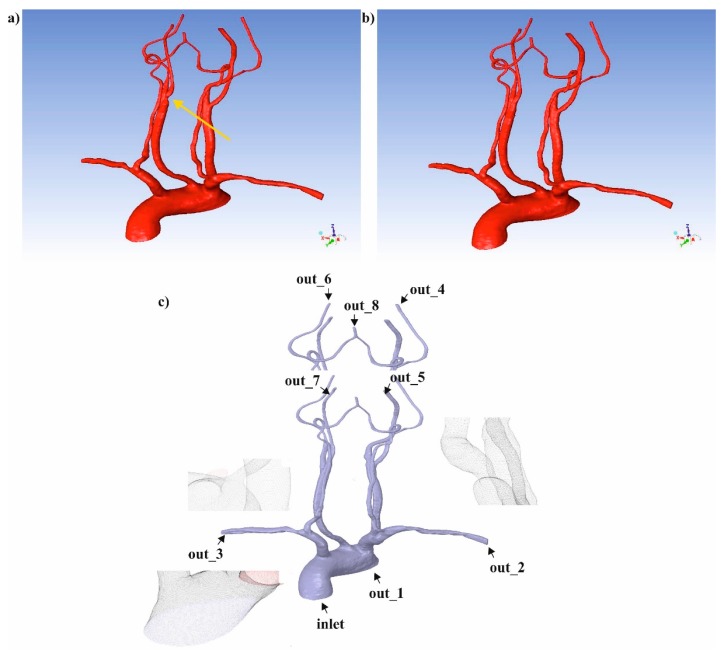
Numerical reconstruction of 3D vascular models representing a part of cardiac system from the aortic arch up to brain arteries (view to the aortic arch from the posterior perspective): (**a**) 3D geometry of analyzed arteries before surgical intervention (yellow arrow indicates place of narrowing); (**b**) 3D geometry of analyzed arteries after surgical intervention; (**c**) an example of a numerical grid generated for the analyzed 3D geometry with marked places of blood inlet and outlet.

In the first step, we made 3D reconstructions of carotid and vertebral arteries before (Figure 3a) and after (Figure 3b) surgical intervention with the use of the self-made semiautomatic algorithm for the image processing and 3DDoctror software (Able Software Corp., Lexington, MA, USA). AngioCT data encompassed vessel from the aortic arch up to the top of the skull. The resolution of the medical images was 512 × 512 and approximately 400 (slices with voxel size of 0.44 × 0.44 × 0.63 mm^3^) were considered. First, AngioCT data had to be manually adjusted for brightness to achieve the highest contrast between blood vessels and surrounding tissues. The region growing technique to extract vessels from the background had to be applied. The self-made semiautomatic algorithm reconstructed small gaps in the blood vessels due to large brightness intensity variation inside the vessel. These gaps were eliminated manually using the ImageJ software and its tool for morphological holes filling. Finally, 3DDoctor software was applied to build 3D virtual models of analyzed vessels.

In the second step, to mimic clinical conditions at the inlet of analyzed mathematical domains, velocity profiles as a function of time including one whole cardiac cycle from US-Dopplers were applied. In the CFD technique first, the pre-processor ANSYS ICEM CFD (ANSYS, Canonsburg, PA, USA) to generate and discretize 3D geometries (Figure 3c) was used. The numerical grids were composed of approximately 2,000,000 tetrahedral elements. Moreover, a boundary layer next to the wall was applied. To neglect the influence of the size and/or number of numerical grid elements on the results of computer simulation, a mesh independent test was performed. Finally, ANSYS FLUENT 18.2 software (ANSYS, Canonsburg, PA, USA), using Euler method for solving Navier-Stokes equations, was applied for blood hemodynamics reconstruction in the analyzed domains as previously described [22]. We assumed that the blood flow was incompressible and laminar and used Dirichlet conditions for the description of the mathematical domain. According to it, the following boundary conditions were applied: domain inlet was described with the use of velocity-inlet (v(x,y,z)), outlets from the domain were described with the pressure conditions, and wall was treated as a rigid structure. Moreover, for the boundary conditions we used the following initial values: as blood velocity profile at the inlet US-Doppler traces before and after balloon angioplasty of the narrowed part of vessel, and at the outlets routine blood pressure value for the certain vessel type. Rheological properties of a blood were described with the use of modified Quemada’s model, as previously described [23,24]. Application of this model allowed to treat blood viscosity not as a constant value, which means that when shear rate is increasing the value of blood viscosity is decreasing. Moreover, Quemada’s model includes initial parameters such as hematocrit (Hct), which was around 40% in the described patient. Therefore, blood hematocrit included in CFD model for the analyzed patients was 40%.

Into the analysis, we included not just the left internal carotid artery (LICA) that was stenotic but also the remaining vessels branching from the aortic arch. Since they create a system of interconnected vessels supplying the brain, pressures depend on each other. Moreover, as the two vertebral arteries join together into the basilar artery, we analyzed them together.

As the paper is focused on the prediction of the hemodynamics after restoration of the blood flow through the critically stenotic artery, we used the initial model (before the surgery) to compute probable models of blood flow through the dilated vessel (after the surgery). Nevertheless, balloon angioplasty of the narrowed artery does not guarantee restoration of the full patency. In this procedure, balloon is introduced through the endovascular catheter and when placed in the narrowed segment it is inflated, extending the vessel’s wall. Atherosclerotic plaque is compressed against the wall and the lumen of the artery increases. In this case it was 80% of the normal diameter. Thus, we used our model to predict three hemodynamic conditions associated with restoration of (1) partially improved (80% of lumen diameter—result actually obtained after the surgery); (2) impaired (60% of lumen diameter) and (3) fully improved blood hemodynamics (100% of lumen diameter). This was translated into changes of pressure. Firstly, based on the medical data from the analyzed patient we reconstructed blood flow for several clinical conditions (one before and three after surgical intervention). Therefore, in postsurgical status routine, blood pressure was set as an average value at the outlets of analyzed mathematical domains (marked as 0% pressure—Table 1). Next, by increasing of blood pressure in the cranial part, impaired blood hemodynamics was simulated (marked as +20% pressure—Table 1). Finally, by decreasing of blood pressure in the cranial part, improved blood hemodynamics was simulated (marked as −20% pressure—Table 1). This approach allowed for analysis of stroke appearance, while higher and lower blood pressure values in the cranial part were calculated.

### 2.3. Mathematical Model Verification

US-Doppler traces and CT-perfusion from the analyzed patient, before and after balloon angioplasty of the narrowed part of vessel, were applied for the process of mathematical model verification. First, US-Doppler traces recorded from the different levels of the analyzed circulatory system were confronted with CFD results. This enabled blood flow character comparison. Each time blood flow rate from CFD simulation for the particular outflow was compared to data from US-Doppler. Next, CT-perfusion around carotid and vertebral arteries was compared with CFD results in the same area. Moreover, the Bland-Altman method was applied to analyze the agreement between medical data and CFD results.

## 3. Results

**Figure 4:**
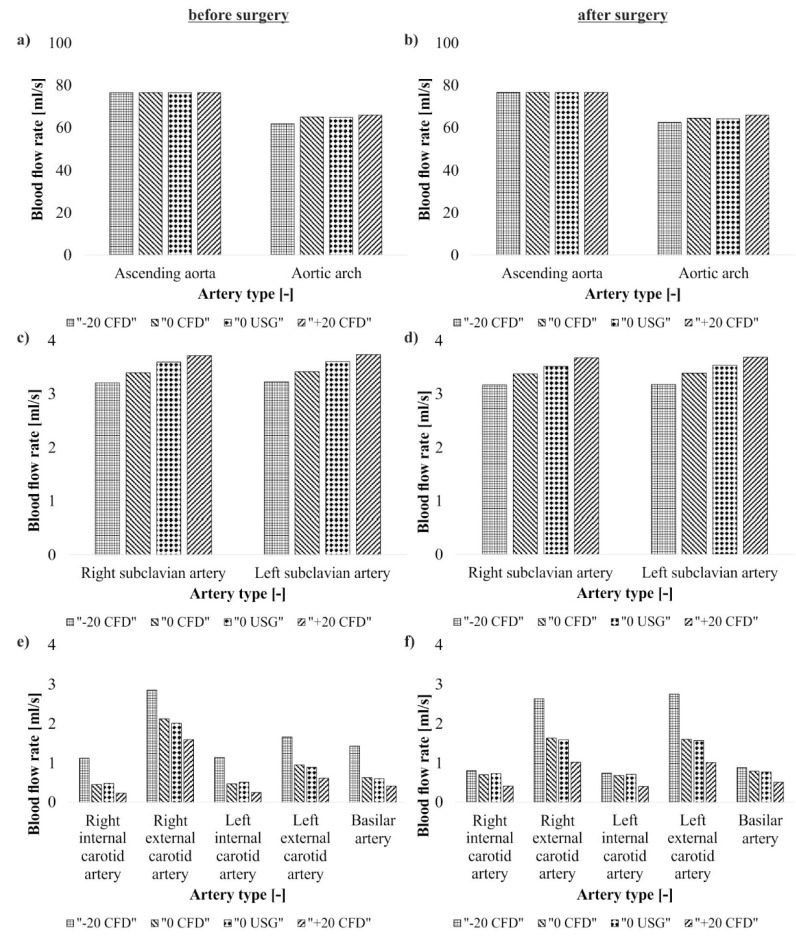
Blood flow rate (**a**) ascending aorta and aortic arch before surgical intervention; (**b**) ascending aorta and aortic arch after surgical intervention; (**c**) left and right subclavian arteries before surgical intervention; (**d**) left and right subclavian arteries after surgical intervention; (**e**) left and right external and internal carotid arteries and basilar artery before surgical intervention; (**f**) left and right external and internal carotid arteries and basilar artery after surgical intervention. Computational fluid dynamics (CFD); US-Doppler (USG).

This section presents a numerical reconstruction of brain ischemic stroke for the patient who experienced a critical stenosis operation in the carotid artery. Different clinical conditions for two stages, before and after balloon angioplasty of the narrowed part of vessel, were analyzed. Firstly, blood perfusion for the clinical condition before and after surgical intervention for aortic arch, subclavian arteries, basilar artery and carotid arteries was analyzed (Figure 4). The overall difference between pre- and post-operation stage amounts to around 10.60% with the highest difference observed for the left external carotid artery (40.63%) and the smallest difference of 0.59% was observed for the right subclavian artery (Table 2). Comparison of CFD and US-Doppler data indicated approximately 0.81% and 1.01% differences for before and after surgical intervention, respectively.

Next, CFD analysis of blood hemodynamics for carotid arteries indicated a 0.62 mL/s increase of blood flow rate in the area of carotid arteries after surgical intervention (from 3.99 mL/s to 4.61 mL/s, respectively) (Table 2). Blood flow rate increased for both internal and left external arteries and decreased for the right external artery after surgical intervention (Table 2). Moreover, comparison of CFD and US-Doppler data indicated approximately 0.47% and 0.98% differences for before and after surgical intervention, respectively.

**Figure 5:**
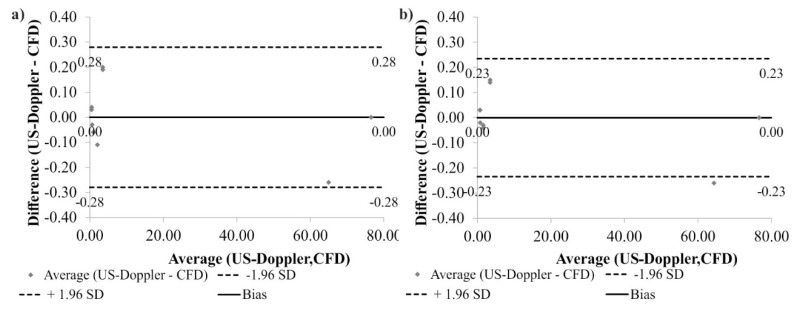
Comparison of computational fluid dynamics (CFD) and US-Doppler data for the analyzed arteries with the use of Bland-Altman analysis: (**a**) before surgical intervention; (**b**) after surgical intervention. Standard Deviation (SD).

Additionally, according to Bland-Altman analysis for all arteries, the difference between CFD and US-Doppler data before surgical intervention was equal to 0.00 mL/s for the range equal to 0.56 mL/s (Figure 5a), and after surgical intervention it was equal to 0.00 mL/s for the range 0.47 mL/s (Figure 5b).

Secondly, blood perfusion for the apparent stroke condition was analyzed. Two cases of higher (+20% pressure) and lower (−20% pressure) blood pressure in analyzed arteries, before and after surgical intervention were analyzed. There was approximately 14.10% and 9.16% differences in blood hemodynamics before and after surgical intervention for higher and lower blood pressure, respectively. The highest difference for the higher blood pressure was observed for the right external carotid artery (55.88%) and the smallest difference of 0.06% was observed for the aortic arch (Table 2). Furthermore, the highest difference for the lower blood pressure was observed for the basilar artery (62.50%) and the smallest difference of 1.01% was observed for the aortic (Table 2).

Next, CFD analysis of blood hemodynamics for carotid arteries for higher blood pressure indicated a 0.16 mL/s increase of blood flow rate in the area of carotid arteries after surgical intervention (2.68 mL/s and 2.84 mL/s before and after surgical intervention, respectively) (Table 2). Blood flow rate increased for both internal and left external arteries and decreased for the right external artery after surgical intervention (Table 2). Meanwhile, for lower blood pressure an increase of 0.15 mL/s of blood flow rate in the area of carotid arteries after surgical intervention was observed (Table 2). Blood flow rate increased for left external artery and decreased for both internal arteries and right external artery after surgical intervention (Table 2).

**Figure 6:**
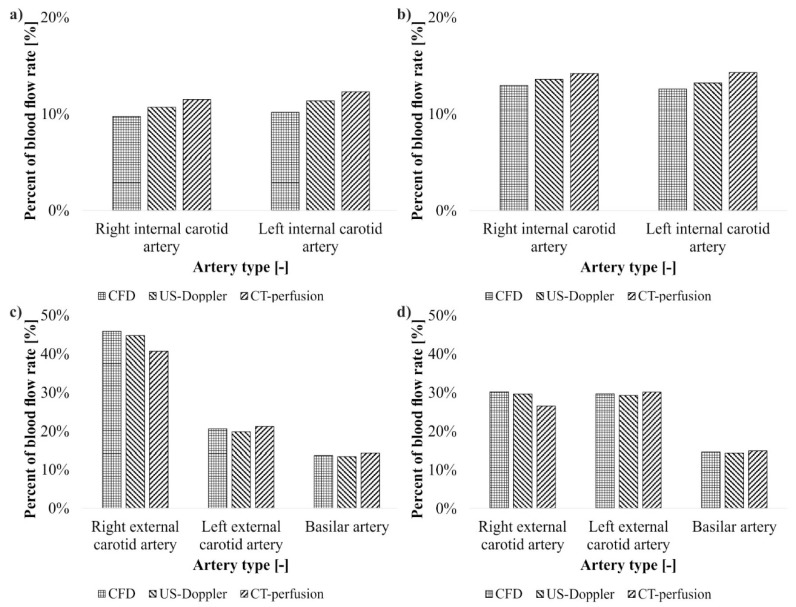
Flow % of blood in the brain for: (**a**) the right and left internal carotid arteries before surgical intervention; (**b**) the right and left internal carotid arteries after surgical intervention; (**c**) the right and left external carotid arteries and basilar artery before surgical intervention; (**d**) the right and left external carotid arteries and basilar artery after surgical intervention. computational fluid dynamics (CFD).

Thirdly, the brain perfusion was analyzed in six separate areas (front, middle and back, on each side) that corresponds with cerebral arteries. Blood flow from CFD simulation and US-Doppler was expressed as percent of blood flow rate and compared to CT-perfusion. It was observed that surgical intervention improved blood flow in the cranium part. Exact values were gathered in Figure 6.

**Figure 7:**
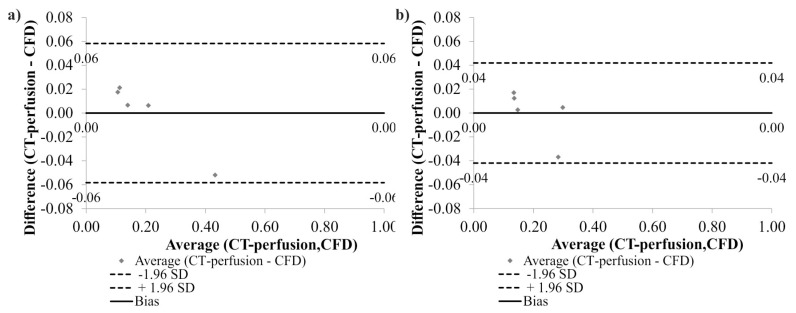
Comparison of computational fluid dynamics (CFD) and computed tomography CT-perfusion data for the analyzed arteries with the use of Bland-Altman analysis: (**a**) before surgical intervention; (**b**) after surgical intervention. Standard Deviation (SD).

Additionally, according to Bland-Altman analysis for all arteries, the difference between CFD and CT-perfusion data before surgical intervention was equal to 0.00 mL/s for the range equal to 0.12 mL/s (Figure 7a), while after surgical intervention it was equal to 0.00 mL/s for the range 0.08 mL/s (Figure 7b).

**Figure 8:**
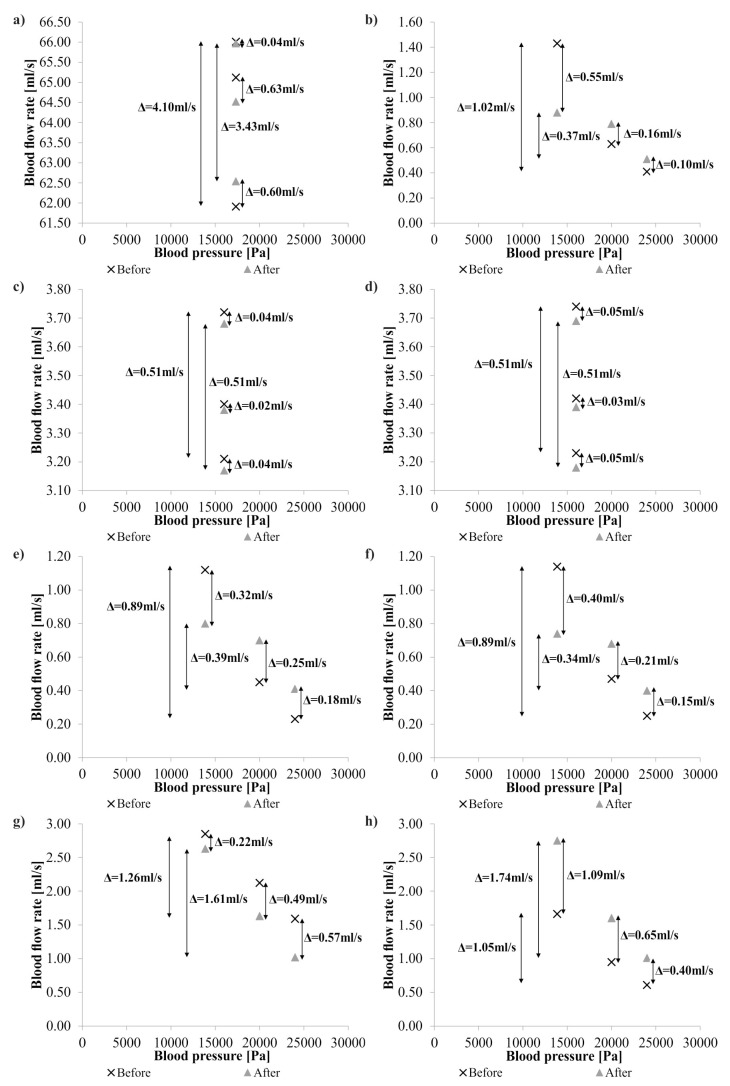
Comparison of blood hemodynamics for different blood pressure before and after surgical intervention: (**a**) aortic arch; (**b**) basilar artery; (**c**) right subclavian artery; (**d**) left subclavian artery; (**e**) right internal carotid artery; (**f**) left internal carotid artery; (**g**) right external carotid artery; (**h**) left external carotid artery.

Finally, it was analyzed if pressure or spatial configuration is a dominant parameter which changes blood hemodynamics (Figure 8). Change of blood pressure in abdominal and subclavian arteries compared to surgical intervention in the left carotid artery indicated a higher change of blood hemodynamics in analyzed arteries. The highest difference in blood hemodynamics was equal to 0.65 mL/s for the clinical conditions (0% pressure). Meanwhile, blood pressure changes (+20% pressure and −20% pressure) caused the highest difference in blood hemodynamics equal to 1.09 mL/s. Moreover, when lower and higher blood pressure were compared, the highest difference in blood hemodynamics was equal to 4.10 mL/s.

## 4. Discussion

In this study, we analyzed an effect of the endovascular treatment of the carotid artery critical stenosis to predict its outcome in the context of brain perfusion. Computational model of blood hemodynamics together with US-Doppler, AngioCT and CT-perfusion data allowed investigation of brain blood distribution. We investigated how different degrees of artery stenosis dilatation may affect the pressure in the analyzed artery. Our results indicated that blood pressure was a crucial parameter, while enlargement of a vessel lumen around narrowness resulted in uniformity of blood flow.

3D models were produced with the use of AngioCT data, processed based on the self-made semiautomatic algorithm together with commercial software for the image processing, where brightness was manually adjusted, to achieve the highest contrast between blood vessels and surrounding tissues. Similarly, Mattes et al. developed an algorithm for the evaluation of follow-up CT scans after endovascular repair [25]. Rouet et al., combined 3D ultrasound and AngioCT to assess the maximum diameter of patients with abdominal aortic aneurysm (AAA) [26]. Meanwhile, Chen et al., (2017) proposed a novel real-time augmented reality framework for minimally invasive surgery that achieves interactive geometric from endoscopic surgery with stereo views. The authors’ framework tracks the movement of the endoscopic camera and simultaneously reconstructs a dense geometric mesh of the minimally invasive surgery scene [27]. Moreover, Lindstrom et al., (2017) proposed a computer method for blood vessel geometries assessment based on shape-fitting algorithms from metric vision. Acoustic images of cross sections of the radial artery were acquired, and medical practitioners used a computer application to measure the wall thickness and nominal diameter of blood vessels [28].

There are no studies dedicated to CFD modeling of stroke formation for the patients with critical stenosis in the carotid artery directed into stroke formation. Numerous studies investigated association between carotid plaques progression and the risk of cerebrovascular events in patients with asymptomatic carotid stenosis [29,30]. Most of the previous studies used standard flow velocity [31]. Liu et al., (2018) used in their studies flow resistances and the inlet flow rate from the literature [32]. Meanwhile, in our research, we applied person-specific inlet flow velocity measured by US-Doppler and CT-perfusion, which allowed us to get more accurate parameters of fluid dynamics. Moreover, different outflow conditions, by manipulating pressure distribution, was kept. It was in line with Schrauwen et al., (2015) who for 10 patient-specific bifurcations set up different outflow conditions, with constant inflow derived from computed tomography perfusion imaging [33]. Jia et al., (2017) took a similar approach; they investigated blood hemodynamics for the stroke case in patients with asymptomatic carotid plaque with the use of MRA technique [34].

In our study we described blood as one-phase fluid model [23] and neglected the existence of solid phases described in the multi-phase fluid model [35,36,37]. The solid phase includes erythrocytes, plasma and leukocytes; therefore it complicates the calculations and is not necessary in our model [38,39,40,41]. Moreover, we observed that prediction of stroke formation is directly associated with blood pressure distribution. Therefore, apparent stroke was simulated with numerically controlled blood pressure. Increase of blood pressure in carotid artery indicated increase of blood amount directed into the cranium, while decrease of blood pressure indicated increase of blood amount in aortic arch. It was in line with Kamangar et al., (2017) who showed that the decrease in blood pressure was found downstream to the stenosis as compared to the coronary artery without stenosis [42].

The CFD model proposed in this study has some limitations. Presented results concern two 3D models (patient before and after surgical intervention) in different clinical and numerical conditions. To increase the reliability of the presented CFD model, different complicity of vessel system should be analyzed. Moreover, as we indicated that the crucial parameter is blood pressure, it is mandatory to investigate not only the influence of vessel’s lumen enlargement rate in function of blood hemodynamics restoration but also the influence of different initial pressure values (higher and lower pressure values in the cranial part) as an initial boundary condition. Furthermore, in order to verify a reliability of the CFD model, the number of analyzed patients should be increased.

## 5. Conclusions

Comparison of CFD results and US-Doppler for the right external arteries indicated comparable blood flow rate after surgical intervention (23.11% and 20.90% decrease of flow rate for CFD and USG-Doppler, respectively). While, comparison of CFD results and US-Doppler for the left external arteries indicated comparable blood flow rate after surgical intervention (40.62% and 43.31% decrease of flow rate for CFD and US-Doppler, respectively).

Application of CFD simulations for blood flow reconstruction for clinical conditions in the circulatory system accomplished on average 98.5% and 98.7% accuracy for CFD results compared to US-Doppler before and after surgical intervention, respectively. Meanwhile, CFD compared to CT-perfusion indicated on average 89.7% and 92.8% accuracy before and after surgical intervention, respectively. Thus, the proposed method might be used as a tool for reconstruction of specific patients’ hemodynamics after operation of critical stenosis in the carotid artery. However, further studies are necessary to confirm its usefulness in clinical practice.

## Figures and Tables

**Table 1:** Pressure values set as outlet boundary condition for the particular outlet. Pressure values measured in [Pa]. The place of the particular outlet boundary condition was presented in Figure 3c.

Artery Type	Boundary Conditions	Pressure (Pa)		
		−20% Pressure	0% Pressure	+20% Pressure
Artery arch	out_1	17,331.60	17,331.60	17,331.60
Right subclavian artery	out_2	15,998.40	15,998.40	15,998.40
Left subclavian artery	out_3	15,998.40	15,998.40	15,998.40
Right external carotid artery	out_4	13,865.30	19,998.00	23,997.60
Right internal carotid artery	out_5	13,865.30	19,998.00	23,997.60
Left external carotid artery	out_6	13,865.30	19,998.00	23,997.60
Left internal carotid artery	out_7	13,865.30	19,998.00	23,997.60
Basilar artery	out_8	13,865.30	19,998.00	23,997.60

**Table 2:** Blood flow values from computational fluid dynamics (CFD) and US-Doppler examination for the particular inlets and outlets. Values measured in mL/s.

Artery Type	Blood Flow (mL/s)							
	CFD—Before Surgical Intervention			CFD—After Surgical Intervention			US-Doppler	
							Before Surgical Intervention	After Surgical Intervention
	−20% Pressure	0% Pressure	+20% Pressure	−20% Pressure	0% Pressure	+20% Pressure	0% Pressure	0% Pressure
Ascending aorta	76.56	76.56	76.56	76.69	76.69	76.69	76.56	76.69
Aortic arch	61.91	65.12	66.01	62.54	64.52	65.97	64.86	64.26
Right subclavius artery	3.21	3.40	3.72	3.17	3.38	3.68	3.60	3.52
Left subclavius artery	3.23	3.42	3.74	3.18	3.39	3.69	3.61	3.54
Right internal carotid artery	1.12	0.45	0.23	0.80	0.70	0.41	0.48	0.73
Right external carotid artery	2.85	2.12	1.59	2.63	1.63	1.02	2.01	1.59
Left internal carotid artery	1.14	0.47	0.25	0.74	0.68	0.40	0.51	0.71
Lest external carotid artery	1.66	0.95	0.61	2.75	1.60	1.01	0.89	1.57
Basilar artery	1.43	0.63	0.41	0.88	0.79	0.51	0.60	0.77
